# Growth performance, nutrients digestibility, intestinal microbiota and histology altered in broilers fed maize- or sorghum-based diets

**DOI:** 10.1080/01652176.2024.2373295

**Published:** 2024-07-01

**Authors:** Aaqil Ahmad, Asad Sultan, Shabana Naz, Naila Chand, Ziaul Islam, Ibrahim A. Alhidary, Rifat Ullah Khan, Samia H. Abdelrahman, Sifa Dai

**Affiliations:** a Department of Poultry Science, Faculty of Animal Husbandry and Veterinary Sciences, The University of Agriculture, Peshawar, Pakistan; b Department of Zoology, Government College University, Faisalabad, Pakistan; c Department of Animal Sciences, Shaheed Benazir Bhutto University Sheringal, Dir Upper, Pakistan; d Department of Animal Production, College of Food and Agriculture Science, King Saud University, Riyadh, Saudi Arabia; e College of Veterinary Sciences, Faculty of Animal Husbandry and Veterinary Sciences, The University of Agriculture, Peshawar, Pakistan; f Central Veterinary Laboratory, Khartum, Sudan; g Department of Pharmaceutical and Life Sciences, Jiujiang University, Jiujiang, China; h Jiujiang Bozheng Institute of Biotechnology Industry, Jiujiang, China

**Keywords:** Sorghum, digestibility, broilers, energy, microbiota

## Abstract

This study aimed to evaluate the effect of varying levels of sorghum-based diets as an alternative to maize in broiler nutrition. A total of 320 one-day-old male Ross 708 broiler chickens were randomly allocated to four treatment groups (5 pens per treatment and 16 birds per pen), comprising a control group with a basal diet and groups receiving sorghum-based diets with 20%, 40%, and 100% maize replacement. The overall weight gain was significantly (*p* < 0.0001) higher in the control group, followed by 20%, 40%, and 100% sorghum replacement. Additionally, overall feed intake was significantly (*p* < 0.01) higher in the 20% sorghum replacement group compared to the control and other groups. Broilers fed sorghum-based diets exhibited a significantly (*p* < 0.01) increased feed conversion ratio. Carcass characteristics showed no significant differences between broilers fed corn and sorghum; however, the digestibility of crude protein and apparent metabolizable energy significantly (*p* < 0.01) increased in the 20% sorghum-corn replacement compared to the 40% and 100% replacement levels. Ileal villus height and width did not differ among the corn-sorghum-based diets, regardless of the replacement percentage. Furthermore, among the cecal microbiota, *Lactobacillus* count was significantly (*p* < 0.041) higher in the 20% corn-sorghum diet compared to the 40% and 100% replacement levels. These findings suggest that replacing corn up to 20% of corn with sorghum in broiler diet positively impact growth performance, gut health, nutrient digestibility, and cecal microbiota in broilers. However, larger replacements (40% and 100%) may have negative implications for broiler production and health.

## Introduction

The continuous expansion of poultry industry has intensified the demand for energy and protein-rich feed ingredients (Tilahun and Worku 2022a, 2022b; Hussain et al. 2023). Corn stands out as the predominant cereal grain globally in poultry feed formulations. However, the fluctuating price and availability of corn, influenced by its alternative use in ethanol production, have spurred interest in exploring alternative feed ingredients, with emerging crops like sorghum gaining attention (Puntigam et al. 2020; Ciurescu et al. 2023).

Sorghum holds a significant position as a primary animal feed grain in both India and globally (Xu et al. 2017; Wilk et al. 2022). It ranks as the third most extensively cultivated cereal in Asia, following rice and maize. While its nutritional content falls slightly behind maize, it proves to be more cost-effective and accessible, particularly in semi-arid regions of Asia and Africa (Manwar and Mandal 2009). The diminished nutritional value of sorghum grain may be attributed to the presence of an insoluble protein fraction. This component potentially encases the starch granules, rendering them inaccessible to the birds (Moss et al. 2020). However, the presence of tannins and phytic acid in sorghum can lead to adverse effects, including hindered growth, decreased feed consumption, suboptimal feed utilization, reduced nutrient absorption, and an elevated occurrence of abnormal leg bone development in broiler chickens (Avila et al. 2021). The growth performance of broiler chickens can be affected by the composition of sorghum. Sorghum is distinct in that it contains kafirin, phytate, and may also have condensed tannin, all of which can potentially have adverse effects on its nutritional properties. Phytate and tannin can form complexes with protein in the digestive system, which leads to reduced protein digestibility and absorption of both dietary and naturally occurring amino acids (Selle et al. 2010a).

Sorghum generally offers a more stable and higher energy content compared to wheat, often at a more favorable price. However, diets based on sorghum have been linked to lower broiler performance compared to those based on wheat (Selle et al. 2010b). A study by Robertson and Perez-Maldonado (2006) found that sorghum performed less effectively than wheat in terms of feed efficiency and breast-meat yield, which are vital factors in sustainable chicken-meat production. Additionally, certain studies have suggested that low levels of tannins may have beneficial effects on gut health (Moritz et al. 2023) and enhance digestive performance in broiler chickens (Huang et al. 2018). Despite these potential advantages, poultry farmers exhibit hesitancy in adopting sorghum-based diets, particularly in the initial week of a broiler’s life (Ciurescu et al. 2023).

The study aimed to assess the impact of substituting corn with low-tannin sorghum in diets with equal energy and protein levels on various aspects of broiler chickens, including growth performance, carcass quality, ilium histological features, ileal nutrient digestibility, and ileal microbiota. The hypothesis was that such substitution would not negatively affect broiler performance, as evaluated through growth performance metrics.

## Materials and methods

The corn and sorghum (Sorghum bicolor var. technicum) used in this study were sourced from the local market. The chemical composition of both the corn and sorghum is detailed in Table 1. The sorghum used in this experiment had a low tannin content of 0.72% catechin equivalent, as per the manufacturer’s specifications.

**Table 1.** Calculated nutrient content of broiler diets formulated with replacement of different level sorghum on maize (g/kg; as fed).

Ingredients g/kg	Sorghum % of maize diets			
	Control	20% sorghum replacement	40% sorghum replacement	100% sorghum replacement
Maize corn	566	425	287	0.00
Sorghum grain	0.00	143	283	566
Soybean meal (46%)	346	344	342	346
Poultry oil	50.2	50.2	50.2	50.2
Dicalium phosphate	17.5	17.4	17.2	17.0
Limestone	9.06	8.28	8.68	8.16
Salt	4.46	4.46	4.28	4.13
Lysine-HCL	2.18	2.20	2.20	2.20
Threonine	1.26	1.26	1.24	1.24
DL-methionine	2.64	2.70	2.80	2.80
Choline-Cl & Coban 90	2.25	2.25	2.25	2.22
Vitamin premix	0.25	0.25	0.25	0.25
Calculated nutrient content				
AME_N_, MJ/kg	13.0	13.0	13.1	13.1
Crude protein	210.5	211.0	209.0	210.0
Lysine	12.6	12.4	12.5	12.6
Methionine	6.0	6.1	6.1	6.1
Threonine	8.5	8.5	8.5	8.5
Tryptophan	2.5	2.4	2.4	2.4
Calcium	8.8	8.7	8.6	8.7
Phosphorus	6.0	6.0	6.1	6.1
Non-phytate phosphorus	0.4	0.39	0.41	0.42

Amount per kg as fed: vitamin A 12,000 I.E.; cholecalciferol 124 μg; vitamin E (as all-rac-α-tocopheryl acetate) 50 mg; vitamin K3 4 mg; vitamin B1 3 mg; vitamin B2 7.5 mg; vitamin B6 (Pyridoxine hydrochloride) 4.5 mg; vitamin B12 0.02 mg; nicotinic acid 69 mg; pantothenic acid 19.5 mg; folic acid 1.95 mg; biotin 0.12 mg; iron (as iron-(II)-sulfate, monohydrate) 67 mg; zinc (as zinc sulfate, monohydrate) 67 mg; manganese (as manganese-(II)-oxide 100 mg; copper (as copper-(II)-sulfate, pentahydrate) 17 mg; iodine (as calcium iodate, anhydrous) 1 mg; cobalt (as alkaline cobalt-(II)-carbonate, monohydrate) 1 mg; selenium (as sodium selenite) 0.45 mg.

### Experimental protocol

A total of 320 one-day-old male Ross 708 broiler chickens, with an initial weight of 43.4 ± 0.91 g, were reared in wire-floored cages (80 × 65 x 45 cm³) under 4 treatments and 5 replicates. They were provided with ad libitum access to both feed and water. The ambient temperature was set at 34 °C initially and gradually reduced as the birds aged, reaching a final temperature of 24 °C by day 35. The relative humidity was maintained between 45 to 55%. All birds received vaccinations for Infectious Bursa Disease on days 14 and 21, and for Newcastle Disease on days 7 and 28. The trial encompassed two phases: a starter phase from day 1 to 21, followed by a finisher phase from day 22 to 35.

### Experimental design and diets

Broiler chickens were randomly assigned to one of four treatment groups (5 replicates), which included the control group receiving a basal diet in pellet form. The remaining treatments involve substituting sorghum with corn, either partially (20% or 40%) or entirely (100%). Details of the experimental diet formulations are provided in Table 1. The diets were formulated to be isonitrogenous and isocaloric, adhering to or surpassing breeder guidelines. The chickens were housed in pens with a wood shavings litter floor (10 cm height) and accommodated in a climate-controlled room following the guidelines outlined in the Ross Broiler Guide. Ambient temperature was kept within thermoneutral range based on the age of the birds. Lighting conditions adhered to EU legislation standards as per EU Council Directive 2007/43/EC.

### Broilers performance

The initial and final body weights were recorded for each feeding phase: 1–21 days, 22–35 days, and for the entire 1–35 days period. Feed intakes were recorded on pen basis, allowing for the calculation of feed conversion ratios (FCR). In cases of bird mortality or culling, their body weights were considered to adjust the FCR calculations accordingly. Daily records were maintained to monitor the incidence of such events.

### Carcass characteristics

Upon concluding the 5-week experiment, two chicks/replicate were randomly chosen from each treatment group and subsequently processed for carcass assessment by cervical dislocation. The sampled birds were humanely slaughtered following established protocols of birds’ welfare. The carcasses (back, breast wing and dressing percentage) along with internal organs including the gizzard, liver, intestines, heart, lungs and kidney were meticulously weighed, recorded, and their weights were expressed as a percentage relative to the live weight of the broiler.

### Gut morphology

The ileum tissue samples, approximately 3.0 cm in length (from Meckel’s diverticulum to the ileo-caecalcolonic junction), were collected from two birds for microscopic assessment at 35 days of age. The samples were washed in a 0.1 M phosphate buffer solution (pH 7.4) and fixed in Bouin solution for 3 days. Afterward, the samples were trimmed to remove torn edges and left for an additional 24 h in the fixing solution. Following the removal of fixing solutions with 70% ethanol, the samples were dehydrated in a graded series of alcohol and cleared in xylol. Four semi-serial sections with 7-cm thickness were placed on each slide. Villi dimensions of the ilium were measured using an image capture and analysis system (Image J, 1.47r). The periodic acid of Schiff staining method was employed for further analysis.

### Sample collection and chemical analysis

At 33 days of age, humane euthanasia was performed using cervical dislocation, followed by the collection of ileal digesta (contents) from five birds per replicate. Careful separation of the intestinal tract from visceral organs ensured clean and accurate collection. Ileal digesta were collected from the Meckel diverticulum to a point just before the junction of the ileocecal and colon. The digesta were gently flushed into 250 ml containers and placed on ice packs for preservation before transport to the laboratory. Subsequently, the digesta samples were subjected to freeze-drying to remove moisture and prevent bacterial growth. Following dehydration through lyophilization, the samples were ground to achieve a particle size of less than 0.1 mm for subsequent analysis. These processed samples were then assessed for gross energy using a UV-Vis Spectrophotometer (ISR-2600 plus) and bomb calorimetry (Chincan, Model Ck-XRY-IA, Ziangjing, China). Apparent metabolizable energy (AME) values were calculated using a specific equation based on the analysis results. The AME (in Kcal/Kg of diet) was calculated as follows:

$$\begin{array}{c} \text{AME}=\;\text{Gross Energy}\;\text{in diet}\;-\\ {}[\text{Gross Energy}\;\text{in digesta}/\text{excreta}\times \\ \left(\begin{array}{l} \left(\text{Marker concentration}\;\text{in diet}\right)/\\ \left(\text{Marker concentration}\;\text{in excreta}/\text{digesta}\right) \end{array}\right], \end{array}$$

where GE denotes gross energy, and Marker represents chromic oxide concentration.

For the determination of nutrient digestibility coefficients, the ground ileal samples (particle size < 0.1 mm) were used. All samples were analyzed in duplicate. An indigestible marker, 0.2% chromic oxide (Cr2O3), was utilized to assess Coefficients of Apparent Total Digestibility. The digestibility coefficients were determined using the formula:

Apparent digestibility = 100 – [100 × (% nutrient in digesta × % Cr in diet)/(% nutrient in diet × % Cr in digesta)], where Cr denotes chromic oxide. Additionally, for the determination of ileal amino acid digestibility, the following formula was used:

Apparent ileal digestibility of amino acids = [1 – (Cr concentration in diet)/(Cr concentration in Digesta) × (Amino acid concentration in digesta)/(Amino acid concentration in diet) × 100], where Cr represents chromic oxide.

### Cecal microbiota evaluation

On day 35 of the study, cecal contents from randomly two broilers/replicate were slaughtered to measure the population of Salmonella, Escherichia coli and Lactobacillus spp. In brief, approximately 1 g of fecal sample was serially mixed in buffered peptone water (1:10) and then cultured on selective agar media for the targeted bacterial species. Lactobacillus spp. were cultured on MRS agar at 37 °C with 5% CO2 for 24 h. Each bacterial population was counted with the help of colony counter, expressing the results as log10 colony-forming units/g (Radwan et al. 2022; Rabie et al. 2022).

### Statistical analysis

The gathered data was subjected to one-way analysis of variance (ANOVA) using the mixed model analysis comprising pen as a random factor nested under the respective treatment group. Repeated measures mixed model analysis was used for parameters measured overtime. Pen was considered as small experimental unit of treatment and control. To discern differences between means, Tukey’s test was employed. Statistical significance was determined at a *p* value below 0.05. In addition, orthogonal contrast was to find the effect of different levels of sorghum diets on studied parameters.

## Results

The results presented in Table 2 indicate that the complete substitution of maize with sorghum had a significant impact on growth performance parameters (BWG, FI, and FCR) across the starter phase (day 1–21), finisher phase (day 22–35), and the overall period (1–35 days). Notably, the control group exhibited higher body weight gain and the best FCR compared to the groups with sorghum replacements. Feed intake during the starter phase increased significantly in the groups receiving 20% sorghum replacement. This trend continued in the finisher phase, where body weight gain was significantly higher in the control group, followed by 20%, 40%, and 100% sorghum replacement, respectively. Feed intake during the finisher phase was significantly higher in broilers with 20% sorghum replacement compared to the other groups. FCR was significantly higher in the sorghum replacement groups and the control group compared to the 40% and 100% replacement groups. Similarly, the final body weight was significantly higher in the control group, followed by 20%, 40%, and 100% sorghum replacement. Feed intake was significantly higher in birds with 20% sorghum replacement compared to the other groups. Consequently, FCR was significantly higher in the control group compared to 20%, 40%, and 100% replacement. Linear and quadratic responses were observed for FI, BWG, and FCR in the starter, finisher phases as well as overall basis. However, no linear or quadratic response was found for livability overall basis.

**Table 2.** Effect of replacing dietary maize by high digestible sorghum on growth performance of broiler chickens.

Performance parameters	Treatments					
	CON	20% sorghum replacement	40% sorghum replacement	100% sorghum replacement	*p* value	Orthogonal contrast
Starter Phase 1–21days)						
BWG (g)	704.25^a^±0.62	690.25^b^±0.05	670.25^c^±0.62	670.25^c^±0.75	0.0001	Linear:0.01 Qudratic:0.01
Feed intake (g)	1212.0^a^±0.40	1276.0^b^±0.24	1214.5^a^±2.10	1221.3^a^±0.25	0.0001	Linear:0.01 Qudratic:0.01
FCR	1.70^b^±0.05	1.80^a^±0.07	1.81^a^±0.09	1.81^a^±0.08	0.0001	Linear:0.01 Qudratic:0.01
Livability %	96.75 ± 0.47	95.00 ± 0.04	94.75 ± 0.25	95.50 ± 0.44	0.080	Linear:0.01 Qudratic:0.01
Finisher Phase (22–35days)						
BWG (g)	1020.10^a^±0.09	995.21^b^±0.098	955.07^c^±0.098	920.80^d^±0.076	0.0001	Linear:0.01 Qudratic:0.01
Feed intake (g)	2183.0^a^±0.87	2186.5^b^±0.74	2185.5^a^±0.64	2184.8^a^±0.85	0.002	Linear:0.01 Qudratic:0.01
FCR	2.140^a^±0.091	2.19^a^±0.091	2.28^b^±0.34	2.37^b^±0.45	0.0001	Linear:0.01 Qudratic:0.01
Livability %	96.25 ± 0.47	95.25 ± 0.39	96.75 ± 0.25	96.25 ± 0.39	0.062	Linear:0.11 Qudratic:0.21
Overall period (1–35days)						
Final weight(g)	1724.35^c^±0.087	1645.13^b^±0.054	1625.31^ab^±0.034	1591.32^a^±0.067	0.0001	Linear:0.01 Qudratic:0.01
Feed intake (g)	3395.0^a^±0.076	3462.8^b^±0.056	3408.0^a^±0.067	3406.0^a^±0.089	0.0001	Linear:0.01 Qudratic:0.01
FCR	1.96^b^±0.076	2.10^a^±0.098	2.09^a^±0.043	2.14^a^±0.078	0.0001	Linear:0.01 Qudratic:0.01
Livability %	95.00 ± 0.091	95.00 ± 0.018	94.50 ± 0.028	94.90 ± 0.018	0.066	Linear:0.34 Qudratic:0.41

Means in rows having different superscripts are significantly different.

The impact of various levels of sorghum replacements on carcass quality at day 35 in broilers is presented in Table 3. The results indicate that the weights of the back, breast, wing, dressing percentage, head, gizzard, intestines, heart, lungs, kidney, and liver did not show any significant differences between the control group and the birds fed with different levels of sorghum replacements. No linear and quadric response was found for the increasing levels of sorghum diets on the carcass characteristics of broilers.

**Table 3.** Effect of replacing dietary maize by high digestible sorghum on the carcass traits (%) of broiler chickens at 35 days of age.

Trait	Control	20% sorghum replacement	40% sorghum replacement	100% sorghum replacement	*p* value	Orthogonal contrast
Back (%)	23.43 ± 0.082	23.02 ± 0.002	23.31 ± 0.032	22.13 ± 0.052	0.062	Linear:0.78 Qudratic:0.61
Breast (%)	34.02 ± 0.024	32.10 ± 0.002	33.13 ± 0.082	32.30 ± 0.042	0.070	Linear:0.55 Qudratic:0.11
Wing (%)	11.92 ± 0.081	11.85 ± 0.023	11.93 ± 0.011	11.18 ± 0.042	0.091	Linear:0.71 Qudratic:0.81
Dressing (%)	70.04 ± 0.008	69.13 ± 0.012	69.40 ± 0.072	69.23 ± 0.032	0.081	Linear:0.54 Qudratic:0.76
Gizzard (%)	2.82 ± 0.008	2.94 ± 0.002	2.86 ± 0.008	2.76 ± 0.008	0.214	Linear:0.88 Qudratic:0.54
Intestine (%)	13.76 ± 0.008	13.73 ± 0.008	13.51 ± 0.004	12.97 ± 0.003	0.062	Linear:0.31 Qudratic:0.61
Heart (%)	0.88 ± 0.008	0.80 ± 0.008	0.72 ± 0.008	0.77 ± 0.008	0.080	Linear:0.71 Qudratic:0.76
Lungs (%)	0.79 ± 0.008	0.78 ± 0.006	0.78 ± 0.008	0.78 ± 0.008	0.11	Linear:0.23 Qudratic:0.81
Kidney (%)	0.76 ± 0.008	0.79 ± 0.006	0.78 ± 0.008	0.74 ± 0.008	0.061	Linear:0.98 Qudratic:0.31
Liver (%)	2.82 ± 0.008	2.86 ± 0.0012	2.83 ± 0.003	2.82 ± 0.002	0.073	Linear:0.88 Qudratic:0.51

Means in rows having different superscripts are significantly different.

Table 4 displays the impact of enzyme supplementation on the villus dimensions of ilium of broilers at day 35, which were fed different levels of sorghum-based diet. Villus height and width were significantly (*p* < 0.01) higher in the control groups compared to the broilers fed different levels of replacement of sorghum. Both linear and quadratic responses were found for each villus parameters.

**Table 4.** Effect of replacing dietary maize by high digestible sorghum on the ileal intestinal morphology of broiler.

Intestinal villus indices	Control	20% sorghum replacement	40% sorghum replacement	100% sorghum replacement	*p* value	Orthogonal contrast
Villi height (µm)	712 ± 0.21^a^	684.0 ± 0.08^b^	698.0 ± 0.23^b^	676.0 ± 0.15^b^	0.0001	Linear:0.01 Qudratic:0.01
Villi width (µm)	36.20 ± 0.09^a^	34.0 ± 0.12^b^	35.80 ± 0.22^b^	34.0 ± 0.09^b^	0.003	Linear:0.01 Qudratic:0.01
Villi crypt depth (µm)	106.0 ± 0.21	103.0 ± 0.171	104.0 ± 0.242	102.0 ± 0.126	0.18	Linear:0.51 Qudratic:0.44
Villus height: crept depth	6.7 ± 0.131	6.6 ± 0.345	6.7 ± 0.175	6.6 ± 0.151	0.120	Linear:0.71 Qudratic:0.88

Means in rows having different superscripts are significantly different.

The information regarding ileal nutrient digestibility and metabolizable energy on day 35 of the experimental period is outlined in Table 5. The data suggests that substituting maize with various levels of sorghum has no significant impact on the digestibility of dry matter and crude fat when compared to a maize-based diet. However, substituting maize with sorghum at a 20% level significantly improved the digestibility of crude protein and apparent metabolizable energy compared to a 40% and 100% replacement of the maize-based diet. Except dry matter, linear and quadratic effects were observed for each parameter.

**Table 5.** Effect of replacing dietary maize by high digestible sorghum on the nutrient digestibility of broiler chickens at 42 days of age.

Digestibility %	Treatments				*p* value	Orthogonal contrast
	Control	20% sorghum replacement	40% sorghum replacement	100% sorghum replacement		
Dry matter	77.60 ± 0.01	74.36 ± 0.01	75.40 ± 0.03	74.60 ± 0.05	0.070	Linear:0.61 Qudratic:0.79
Crude protein	77.43^a^±0.50	77.58^a^±0.31	72.61^b^±0.20	70.70^c^±0.33	0.001	Linear:0.01 Qudratic:0.01
Crude fat	87.50 ± 0.34	87.60 ± 0.33	87.32 ± 0.23	86..45 ± 0.23	0.078	Linear:0.55 Qudratic:0.67
AME (Kcal kg^-1^)	2721.72^a^±0.39	2699.21^b^±0.18	2556.23^c^±0.40	2488.21^d^±0.41	0.0001	Linear:0.02 Qudratic:0.01

Means in rows having different superscripts are significantly different.

The details regarding cecal microbes in broilers fed with a maize-based diet and various levels of sorghum on days 21 and 35 are presented in Table 6. The results indicate that the count of *Lactobacillus* spp was significantly (*p* < 0.05) higher in the control group, followed by a 20% replacement at both 21 and 35 days of the broilers’ age. No significant difference was observed in the *Lactobacillus* count between broilers fed with 40% and 100% replacement of sorghum at both intervals of 21 and 35 days. Linear and quadratic effects were not significantly different for each bacterial species.

**Table 6.** Effect of replacing dietary maize by high digestible sorghum on the cecal microbial counts at days 21 and 28 (log_10_cfu g**^−^**^1^).

Gut microbes	Treatments				*p* value	Orthogonal contrast
	Control	20% sorghum replacement	40% sorghum replacement	100% sorghum replacement		
Day 21						
*Escherichia coli*	6.78 ± 0.03	6.78 ± 0.03	6.79 ± 0.02	6.80 ± 0.02	0.45	Linear:0.61 Qudratic:0.79
*Salmonella*	6.80 ± 0.09	6.81 + 0.04	6.85 ± 0.01	6.80 ± 0.09	0.34	Linear:0.61 Qudratic:0.79
*Lactobacillus spp*	7.18 ± 0.02^a^	6.98 ± 0.03^b^	6.52 ± 0.05^c^	6.38 ± 0.03^c^	0.04	Linear:0.01 Qudratic:0.02
Day 28						
*Escherichia coli*	7.31 ± 0.06	7.38 ± 0.07	7.30 ± 0.05	7.33 ± 0.03	0.90	Linear:0.61 Qudratic:0.79
*Salmonella*	6.88 ± 0.07	6.78 ± 0.06	6.82 ± 0.05	6.81 ± 0.064	0.06	Linear:0.61 Qudratic:0.79
*Lactobacillus spp*	7.25 ± 0.01^a^	7.08 ± 0.02^b^	6.81 ± 0.02^c^	6.54 ± 0.07^c^	0.04	Linear:0.21 Qudratic:0.01

Means in rows having different superscripts are significantly different (*p* < 0.05).

## Discussion

In this study, we investigated the impact of substituting corn with low-tannin sorghum [Sorghum bicolor (L.) Moench] on broiler performance, carcass quality, ilium histology and nutrient digestibility. Our findings revealed significantly higher feed intake, weight gain, and feed conversion on 1 to 21 days and 22 to 35 days periods when corn was replaced at the level of 20%. This suggests that low-tannin sorghum grain can only be replaced only at the level of 20%, beyond this level has no significant effect on broiler performance. Córdova-Noboa et al. (2018) observed a reduction in average daily feed intake (ADFI), leading to a decrease in average daily weight gain (ADWG) and, consequently, body weight (BW). These negative consequences have been attributed to the high tannin content in the sorghum added to the diets in their experiment, which can negatively impact nutrient digestion (del Puerto et al. 2016). However, Torres et al. (2013), Manyelo et al. (2019), Puntigam et al. (2020) and Ciurescu et al. (2023) concluded that the partial or total replacement of corn with low-tannin sorghum did not negatively affect performance measurements in broilers. Nonetheless, Manyelo et al. (2019) found that replacing maize with sorghum at levels of 25%, 50%, 75%, and 100% led to progressively higher body weight gain and improved Feed Conversion Ratio (FCR), with no discernible impact on feed intake.

Contrary to the growth performance, carcass parameters did not change significantly between the control and the sorghum. These findings on carcass traits align with studies by Thomas and Ravindran (2008), Pasquali et al. (2016), Saleh et al. (2019), da Silva et al. (2018), El-Afifi et al. (2013), and George et al. (2017).

In the current study, digestibility of crude protein was significantly higher in broilers fed with 20% replacement of sorghum and similar to corn-based diet. Additionally, AME observed in 20% replacement of sorghum-based diets was greater compared to 40 and 100% corn replacement. The higher digestibility of crude protein and AME could partially be attributed to a longer feed retention time, as evidenced using 20% sorghum-based diets. However, this effect was diminished when the level of sorghum was increased in the diet. Furthermore, sorghum may exhibit relatively lower digestibility coefficients for starch compared to maize. Ingredients with such characteristics, characterized by limited starch digestion, tend to reside in the small intestine of birds for extended durations (Weurding et al. 2001). The diminished digestibility of sorghum protein at higher levels has been linked to karifin’s poor digestibility and the binding of condensed tannins with dietary proteins (Hagerman et al. 1998; Wong et al. 2010).

In the present study, there was a notable reduction in both villus height and width across the sorghum-replaced groups, regardless of the replacement level. However, villus crypt depth and the ratio of villus height to crypt depth exhibited no significant changes among the groups. These outcomes bear partial resemblance to the observations made by Manyelo et al. (2019), who reported no significant differences in ileal dimensions among broiler chickens subjected to various levels of corn replacement with sorghum. Silva et al. (2015) and Fernandes et al. (2008) also observed no differences in villi heights and crypt depths in broiler chickens. Nyamambi et al. (2007) observed a decline in villi heights and crypt depths in broiler chickens when maize meal was substituted with sorghum meal in diets varying in condensed tannin levels. The authors suggested that the decrease in villi height and crypt depth was attributed to the presence of condensed tannins, which may have caused damage to the intestinal villi.

The gut microflora plays a dual role, functioning as both a potential energy source through fermentation and offering numerous advantages for gut health. However, in broiler chickens, the intestinal microflora also competes with the host for nutrients within the digestive tract (Gabriel et al. 2003). According to Wu et al. (2017), birds fed wheat exhibited a tendency to have higher Lactobacillus counts compared to those fed sorghum. This observation extended to an inclination for wheat-fed birds to display greater Lactobacillus colonization and lower total gut bacterial counts in contrast to birds fed sorghum. The increased presence of Lactobacillus is potentially advantageous, as Lactobacillus species are known to produce digestive enzymes that facilitate feed conversion efficiency and digestion in host animals (Ramaswami et al. 2005). Moreover, studies have reported the inhibitory effect of Lactobacillus on the proliferation of pathogenic bacteria, including, *E. coli, Salmonella and Clostridium*, through competitive exclusion (Kawai et al. 2004). Fagundes et al. (2017) concluded that the substitution of corn with sorghum in broiler diets results in a modification of the intestinal microbiota. This alteration is characterized by a reduction in Clostridium counts and an increase in the Lactobacillus population in both the small intestine and caeca. Importantly, this shift in microbiota composition did not adversely affect the performance of broilers.

## Conclusion

In conclusion, substituting up to 20% of corn with sorghum in broiler diets positively influenced the growth without compromising carcass characteristics. However, higher replacement levels (40% and 100%) showed potential negative effects on feed conversion and cecal microbiota, emphasizing the need for careful consideration in formulating optimal sorghum inclusion levels for broiler nutrition.

## Data Availability

This data are available in student thesis.
